# *RyR1*-related myopathy mutations in ATP and calcium binding sites impair channel regulation

**DOI:** 10.1186/s40478-021-01287-3

**Published:** 2021-11-22

**Authors:** Qi Yuan, Haikel Dridi, Oliver B. Clarke, Steven Reiken, Zephan Melville, Anetta Wronska, Alexander Kushnir, Ran Zalk, Leah Sittenfeld, Andrew R. Marks

**Affiliations:** 1 Department of Physiology and Cellular Biophysics, Vagelos College of Physicians and Surgeons of Columbia University, New York, NY 10032 USA; 2 Department of Anesthesiology, Vagelos College of Physicians and Surgeons of Columbia University, New York, NY 10032 USA; 3 Wu Center for Molecular Cardiology, Vagelos College of Physicians and Surgeons of Columbia University, Russ Berrie Medical Science Pavilion, 1150 St. Nicholas Avenue, New York, NY 10032 USA; 4 Ilse Katz Institute for Nanoscale Science and Technology, Ben Gurion University of the Negev, Beersheba, Israel

## Abstract

The type 1 ryanodine receptor (RyR1) is an intracellular calcium (Ca^2+^) release channel on the sarcoplasmic/endoplasmic reticulum that is required for skeletal muscle contraction. RyR1 channel activity is modulated by ligands, including the activators Ca^2+^ and ATP. Patients with inherited mutations in RyR1 may exhibit muscle weakness as part of a heterogeneous, complex disorder known as *RYR1*-related myopathy (*RYR1*-RM) or more recently termed RYR1-related disorders (RYR1-RD). Guided by high-resolution structures of skeletal muscle RyR1, obtained using cryogenic electron microscopy, we introduced mutations into putative Ca^2+^ and ATP binding sites and studied the function of the resulting mutant channels. These mutations confirmed the functional significance of the Ca^2+^ and ATP binding sites identified by structural studies based on the effects on channel regulation. Under normal conditions, Ca^2+^ activates RyR1 at low concentrations (µM) and inhibits it at high concentrations (mM). Mutations in the Ca^2+^-binding site impaired both activating and inhibitory regulation of the channel, suggesting a single site for both high and low affinity Ca^2+^-dependent regulation of RyR1 function. Mutation of residues that interact with the adenine ring of ATP abrogated ATP binding to the channel, whereas mutating residues that interact with the triphosphate tail only affected the degree of activation. In addition, patients with mutations at the Ca^2+^ or ATP binding sites suffer from muscle weakness, therefore impaired RyR1 channel regulation by either Ca^2+^ or ATP may contribute to the pathophysiology of *RYR1*-RM in some patients.

**Supplementary Information:**

The online version contains supplementary material available at 10.1186/s40478-021-01287-3.

## Significance

The ryanodine receptor/calcium release channel (RyR1) is required for skeletal muscle excitation–contraction (EC) coupling. Mutations in RyR1 that render the channel leaky (unable to close properly) to calcium (Ca^2+^) cause an inherited form of muscle weakness known as RyR1-related disorders (RyR1-RD). Using the high-resolution RyR1 structure solved in our laboratory we identified binding sites for the channel activators Ca^2+^ and ATP. Mutagenesis of these sites combined with functional studies confirmed that they are indeed the key ligand binding sites. Both the ATP and Ca^2+^ sites are involved in disease-causing mutations that alter the response of the channel to these physiological activators and likely contribute to the pathophysiology of RyR1-RD.

## Introduction

Calcium is a vital second messenger [6, 38] that regulates numerous cellular signaling pathways, including muscle contraction [38], hormone secretion [57], and synaptic transmission [64]. Ryanodine receptors (RyRs) are located on the sarcoplasmic/endoplasmic reticulum (SR/ER) and mediate the release of Ca^2+^ from intracellular stores [56]. The three mammalian isoforms, RyR1, RyR2, and RyR3, share approximately 70% sequence identity. RyR1 and RyR2 are widely expressed and are the major SR Ca^2+^ release channels in skeletal and cardiac muscles, respectively [54, 77]. RyR3 was originally found in the brain, but it is also expressed in other tissues [58].

RyR1 is required for excitation–contraction (EC) coupling in skeletal muscle. RyR1 is a homotetramer comprised of four 565 kDa protomers and as such is the largest known ion channel. In addition, regulatory and targeting proteins for enzymes including protein kinase A (PKA) and CaM kinase II (CAMKII), are associated with the channel and regulate its function [41, 45].

*RYR1*-related myopathies (*RYR1*-RM), or as recently proposed *RYR1*-related disorders (*RyR1*-RD) [31], are rare, inherited disorders, the prevalence of which have likely been underestimated at 1:90,000 individuals [1]. Indeed, RYR1-RD is the most common form of non-dystrophic muscle disease and includes individuals with malignant hyperthermia susceptibility that affects ~ 1:3000–1:8500 and possibly as many as 1 in 400 [31]. RYR1-RD exhibits both autosomal dominant and recessive inheritance as well as de novo occurrences. RYR1-RD is characterized by pleotropic clinical presentations ranging from mild to severe muscle weakness, and moderate to severe respiratory insufficiency, which is more often apparent in recessive cases. Some mutations in *RYR1* (19q13.2) result in leaky channels that promote muscle weakness and damage in *RyR1*-RD patients [27]. Although there are currently no approved treatments, a clinical trial using a novel Rycal drug that fixes the leak in RyR1 channels is currently underway at the NIH (NCT04141670).

The RyR1 macromolecular complex includes calstabin [43, 45, 69, 74], PKA [52], CaMKIIδ [26, 70], the phosphatases PP1 and PP2A [15, 42], the phosphodiesterase PDE4D3 [32, 57], sorcin [19], calmodulin [49, 51, 53, 66, 78], triadin [55], junction [33], and calsequestrin [4]. RyR channels are regulated by posttranslational modifications including phosphorylation [27, 44, 45], oxidation [2, 59, 60], and nitrosylation [5]. RyR channels exhibit a bell-shaped response to cytosolic Ca^2+^, with activation at micromolar levels and inhibition at millimolar concentrations [7]. ATP is a potent activator of RyR [48] and millimolar ATP concentrations enhance Ca^2+^-dependent activation of RyR1, manifested as increased open probability (Po) [8, 17, 30, 50, 63, 65]. In disease states, RyR channels may exhibit a stressed-induced leak that contributes to the pathophysiology of heart failure [22, 39, 40, 45], cardiac arrhythmias [69], diabetes [57], muscular dystrophy [3], age-dependent loss of muscle function [2], cancer-associated muscle weakness [68], post-traumatic stress disorder [36], Alzheimer’s Disease [9, 28], and Huntington’s Disease [16].

**Fig. 1:**
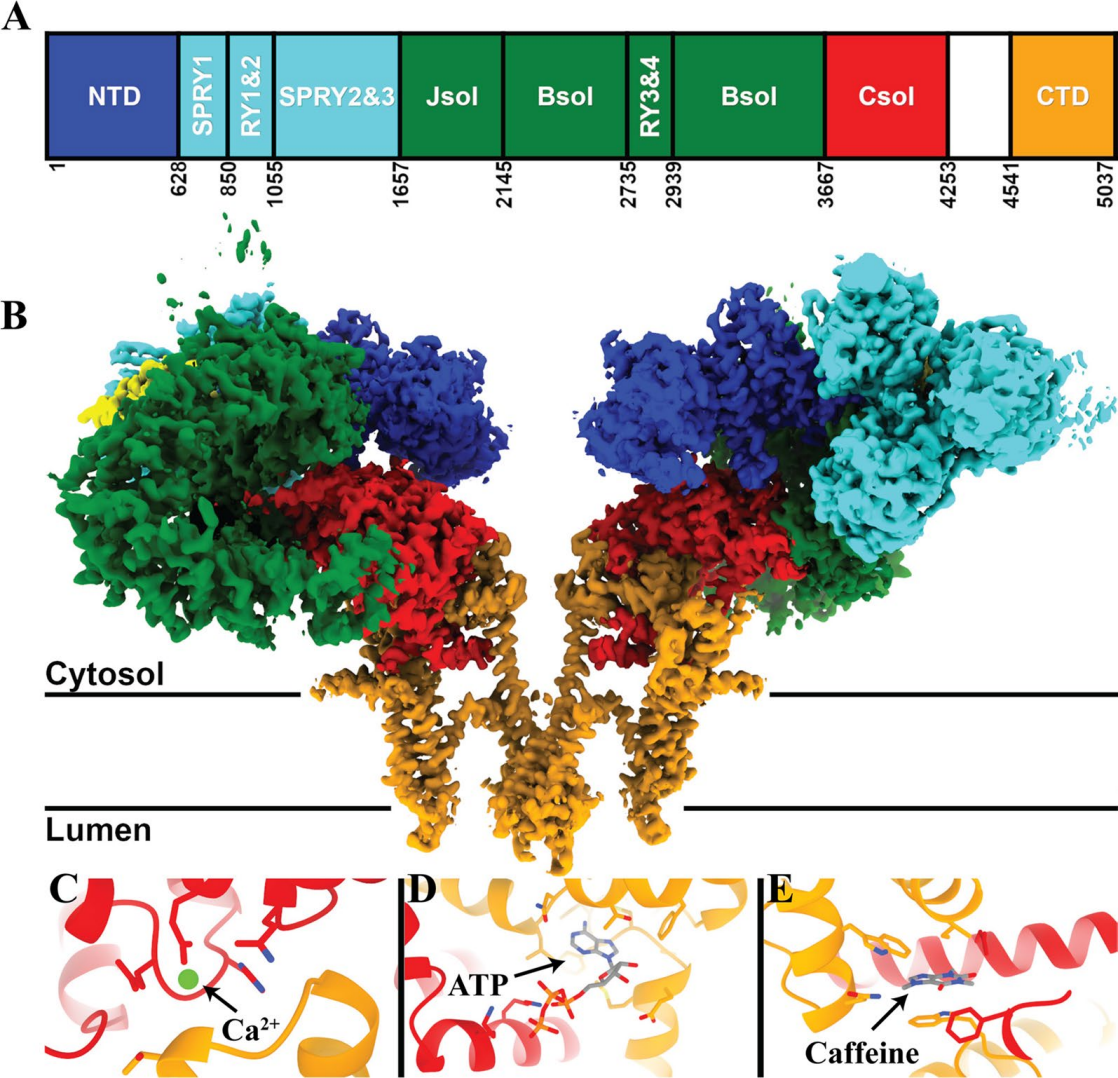
Architechture of RyR1 channel. **A** Schematic diagram of the domain architecture of RyR1. **B** Coulombic density map of RyR1 (PDBID: 7M6A). The accessory protein calstabin-1 is shown in yellow. Zoom in showing Calcium (**C**), ATP (**D**), and caffeine (**E**) binding sites with the coordinating residues [14, 76]

In 2015, three cryogenic electron microscopy (cryo-EM) studies, including our own, described the high-resolution architecture of the closed state of RyR1 [18, 73, 76], revealing that RyR1 belongs to the six transmembrane (6TM) cation channel family. As opposed to most members of the 6TM family, RyR is not voltage gated; however, it contains an evolutionarily conserved pseudo-voltage-sensor domain (pVSD) [76] which lacks the positively charged residues present in voltage-gated channels. We also solved the structure of the open state of RyR1, activated by Ca^2+^*,* ATP, and caffeine, revealing the structural basis of channel gating and ligand-dependent activation of RyR1 [14]. The cytosolic shell of RyR is composed of alpha-solenoid repeats, including two N-terminal beta-trefoil domains (NTD-A and NTD-B) [67], three SPRY domains (SPRY1-SPRY3) [29], two pairs of RYR repeats (RY1&2 and RY3&4) [61, 75], and a pair of EF-hands (EF1&2) [71] inserted in the core solenoid [76]. The activation domain contains a thumb and forefinger motif (TaF), which clamps the zinc finger-containing C-terminal domain (CTD) and provides allosteric coupling between the movement of the cytosolic shell and dilation of the pore aperture. The core solenoid (C-Sol), which is part of the activation domain, links the pore domain to the shell. Binding sites for Ca^2+^, caffeine, and ATP were identified at interdomain interfaces of the C-terminal domain and the transmembrane domain [14] where they likely stabilize interdomain interactions and amplify the effects of Ca^2+^ binding on the gating of the channel pore (Fig. 1).

Our previous work examined the pathophysiological mechanisms underlying RyR1-RD [27]. The present study extends these observations at the atomic level by focusing on structure/function studies of the ATP and Ca^2+^ binding sites and disease causing mutations that affect these sites in patients with RyR1-myopathy. In the present study, we used site-directed mutagenesis to assess the functional importance of the ATP and Ca^2+^ binding sites that we previously identified using cryo-EM. Moreover, RyR1 channel mutations found at the Ca^2+^ and ATP binding sites of patients with *RYR1*-RD resulted in defective regulation by Ca^2+^ and ATP that may contribute to muscle weakness in *RYR1*-RD patients.

## Results

### Architecture and function of the RyR1 ATP binding site

**Fig. 2:**
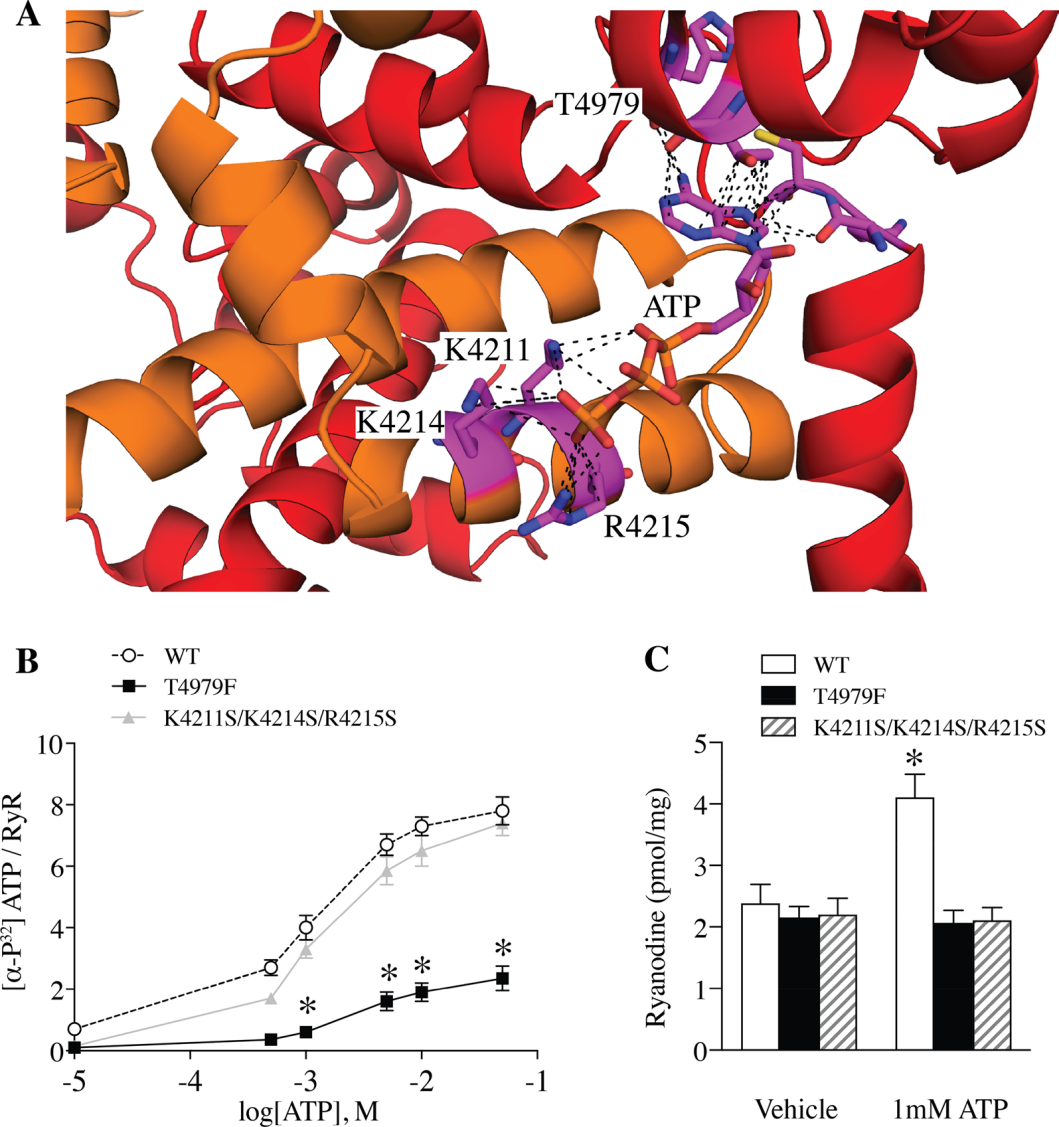
Structure of the ATP binding site. **A** The adenine ring binding site (T4979) and triphosphate tail interacting residues (K4211, K4214, and R4215) are labeled and depicted in stick representation. **B** [α-32^P^]-ATP/[3^H^]-ryanodine binding to ER microsomes of HEK293 cells expressing WT RyR1, T4979F, and K4211S/K4214S/R4215S. **C**, [^3^H]-ryanodine binding to ER microsomes of HEK293 cells expressing WT RyR1 and ATP binding site mutants in response to ATP. Data are presented as the mean ± S.E.M. N = 3 for each group. **P < 0.01

The ATP binding site of RyR1 is located at the junction of the cytoplasmic extension of S6 (S6c) tranmembrane helix and the CTD [14]. Based on the structure, T4979 of the CTD contributes to the adenine ring binding site, and the positively charged K4211, K4214, and R4215 residues of the TaF interact with the triphosphate tail of ATP (Fig. 2A). Based on this model, we hypothesized that mutation of T4979 would reduce ATP binding to RyR1, whereas mutation of K4211, K4214, and R4215 would reduce ATP-dependent activation of RyR1, since ADP and AMP are less effective activators of RyR1 [10, 30].

To assess the functional effects of the ATP binding site. T4979 was mutated to phenylalanine (T4979F), near the adenine ring of ATP, and a triple mutant of K4211/K4214/R4215, each to serine, near the triphosphate tail of ATP was generated. These mutants were made in recombinant rabbit RyR1 and expressed in HEK293 cells. To assess the effects of these mutations, [α-^32^P]-ATP binding, [^3^H]-ryanodine binding, which is a surrogate measure of channel activity, and single channel recordings in planar lipid bilayers were performed. The RyR1 T4979F mutant channel exhibited reduced [α-^32^P]-ATP binding, with a Bmax of 2.5 ± 0.25 pmol ATP/pmol RyR1 compared to a Bmax of 8.0 ± 0.77 pmol ATP/pmol RyR1 for WT RyR1. These data confirm that T4979 is a critical residue for ATP binding to RyR1. Furthermore, the Bmax of 8 suggests that there is likely a second ATP binding site in RyR1 with two molecules of ATP are binding per protomer of the homotetrameric channel. In contrast, the K4211S/K4214S/R4215S mutant channel exhibited normal [α-^32^P]-ATP binding to RyR1 (Fig. 2B), indicating that the complete triphosphate tail is not required for ATP binding to RyR1.

We then used [^3^H]-ryanodine binding to further evaluate activation of RyR1 by ATP binding. Ryanodine binds to pore of the RyR1 channel in the open state [12, 14] and therefore binding can be used as an indicator of channel activity. In the absence of ATP, the levels of [^3^H]-ryanodine binding to T4979F RyR1 and K4211S/K4214S/R4215S RyR1 were similar to that of the WT RyR1 (Fig. 2C). In contrast, in the presence of 1 mM ATP, WT RyR1 exhibited significantly increased [^3^H]-ryanodine binding indicating channel activation, whereas T4979F and K4211S/K4214S/R4215S mutant channels did not (Fig. 2C). Thus, the ATP binding site identified by cryo-EM [14] is a functional site that regulates activity of RyR1.

**Fig. 3:**
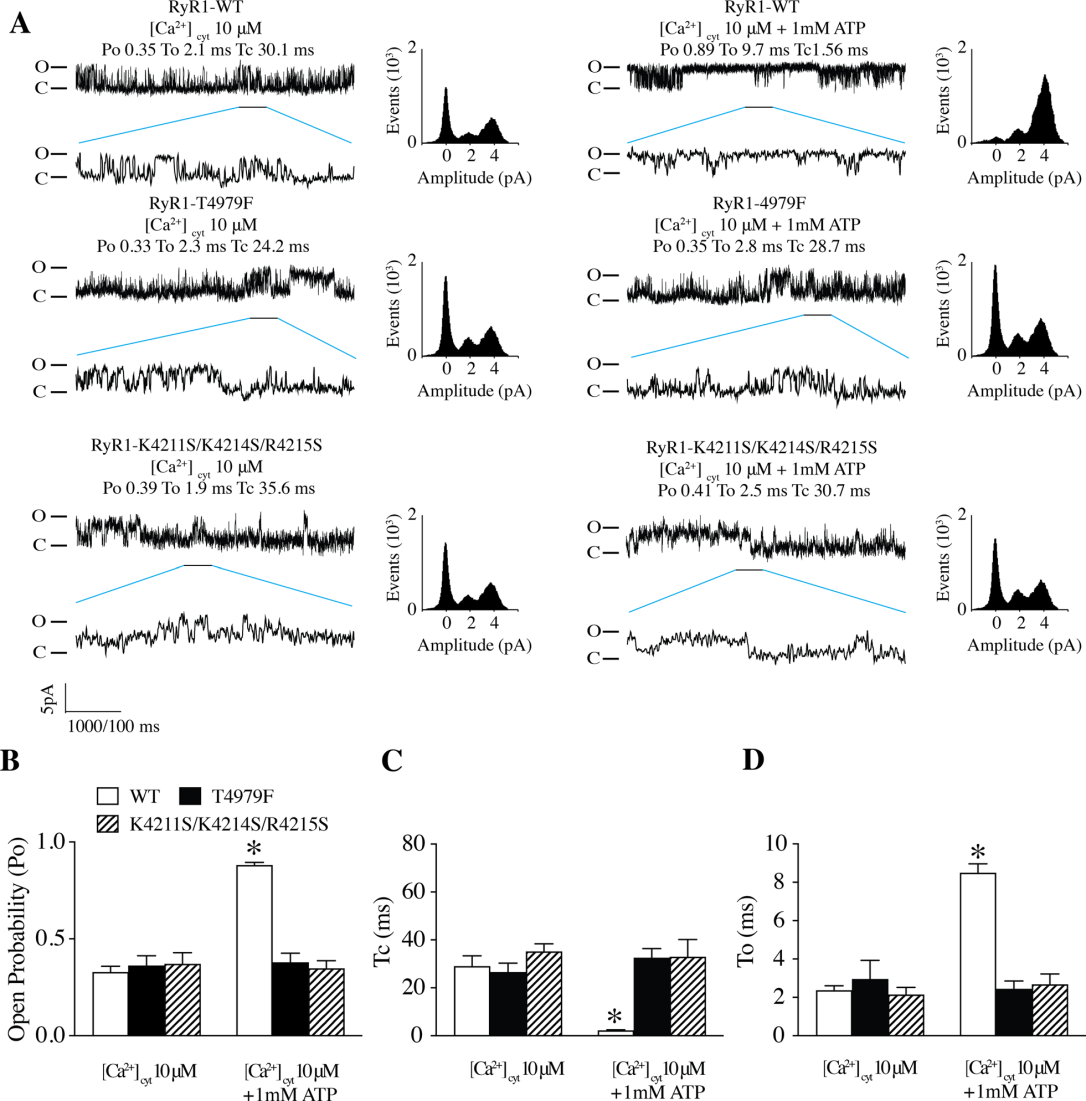
Effects of ATP binding site mutations on the gating of RyR1 channels. **A** Representative single channel traces of WT RyR1 (top), T4979F (middle), and K4211S/K4214S/R4215S (bottom) under 10 µM Ca^2+^ only (left) or 10 µM Ca^2+^ and 1 mM ATP (right). **B** Po, **C** To, and **D** Tc of single WT RyR1, T4979F, and K4211S/K4214S/R4215S mutants. Data are presented as mean ± S.E.M from 6 single channels for each group. *P < 0.05

Previously, we reported that in the presence of 30 µM [Ca^2+^]_*cyt*_, the open probability of WT RyR1 channels was ~ 20%, while in the presence of Ca^2+^/ATP/caffeine (30 µM, 1 mM, 2 mM), the open probability was ~ 90% [14]. This finding is consistent with many previous reports from multiple laboratories [47]. Single channel recordings were used as an additional assessment of the activation of RyR1 by ATP. WT RyR1 channels exhibited an open probability (Po) of 20%, mean open time (To) of 2.1 ms and mean closed time (Tc) of 30.1 ms at 10 µM [Ca^2+^]_*cyt*_. Mutant RyR1 channels T4979F and K4211S/K4214S/R4215S exhibited similar single channel properties compared to WT RyR1 under this condition, suggesting normal Ca^2+^ dependent activation. Addition of 1 mM ATP dramatically increased WT RyR1 Po (Fig. 3A, B), with increased To and reduced Tc of single RyR1 channels (Fig. 3A, C, D). However, 1 mM ATP had no effect on the Po of T4979F or K4211S/K4214S/R4215S mutant channels (Fig. 3A–D). These data further indicate that these mutations eliminate ATP-dependent activation of RyR1 and confirm the functional importance of the ATP binding site idenbtified using cryo-EM (Fig. 2C).

### Architecture and function of the RyR1 Ca^2+^ binding site

**Fig. 4:**
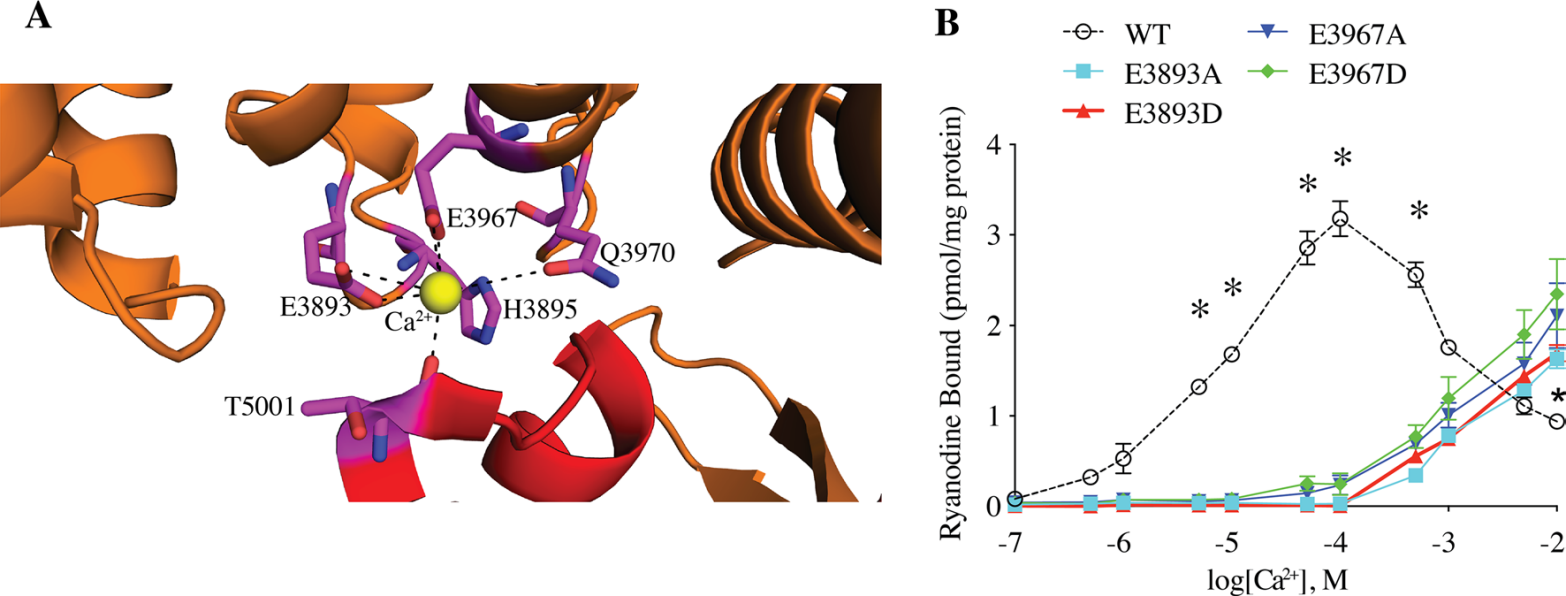
Structure of the calcium binding site. **A** ribbon structure of the Ca^2+^ binding site showing interacting residues. **B** [^3^H]-ryanodine binding to ER microsomes of HEK293 cells expressing WT RyR1 and calcium binding site mutants. Data are presented as mean ± S.E.M from 6 single channels for each group. **P < 0.01

Comparison of difference maps calculated from RyR1 preparations with or without 30 μM Ca^2+^ revealed a Ca^2+^ binding site located at the interface of the CTD and the C-Sol [14]. This Ca^2+^ binding site is primarily comprised of five amino acids (Fig. 4A) which are conserved between RyR and the homologous inositol trisphosphate receptor (IP3R) channels [14, 21]. E3893 and E3967 from the C-Sol and T5001 from the CTD directly interact with Ca^2+^, and H3895 and Q3970 from the C-Sol indirectly interact with Ca^2+^ (Fig. 4A). In order to assess Ca^2+^-dependent ryanodine binding, we mutated E3893 and E3967 to either alanine or aspartic acid, expressed each mutant in HEK293 cells, and then determined [3H]-ryanodine binding to isolated ER vesicles. As shown in Fig. 4B, WT RyR1 exhibits a bell-shaped Ca^2+^ response with peak activation at 100 μM Ca^2+^. In contrast, E3893A, E3893D, E3967A, and E3967D mutant RyR1 channels exhibited both impaired activation at low [Ca^2+^] and impaired deactivation at high [Ca^2+^] (Fig. 4B).

**Fig. 5:**
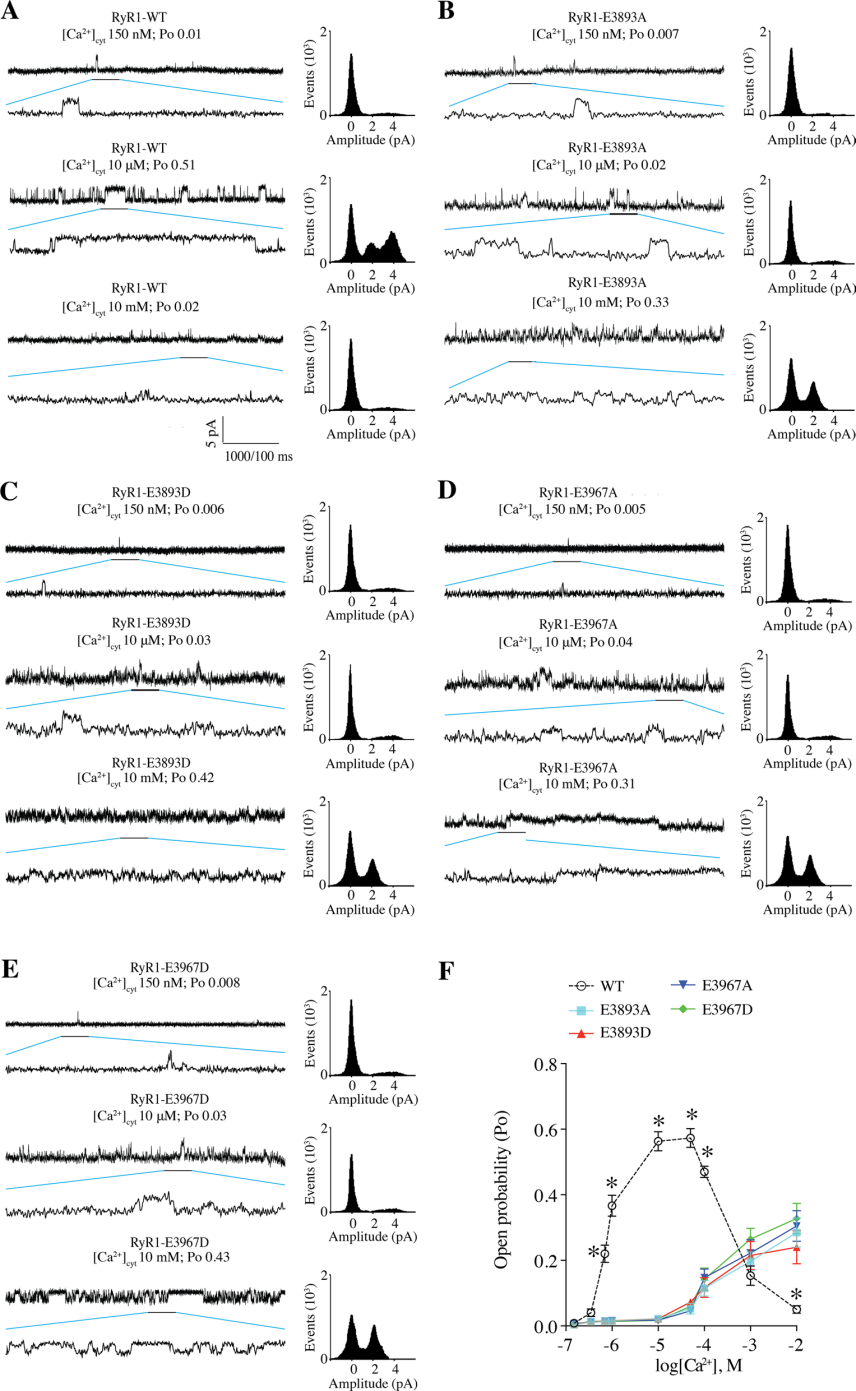
Effects of mutations in the Ca^2+^ binding site on the gating of RyR1 channels. Representative single channel traces of WT RyR1 (**A**), E3893A (**B**), E3893D (**C**), E3967A (**D**), and E3967D (**E**), under 150 nM Ca^2+^, 10 µM Ca^2+^, or 10 mM Ca^2+^. F, Po versus Ca^2+^ concentration curve. Data are presented as mean ± S.E.M from 6 single channels for each group

To further assess the role of the Ca^2+^ binding site in RyR1 channel gating, we examined the single channel properties of the E3893A, E3893D, E3967A, and E3967D RyR1 channel mutants. WT and Ca^2+^-binding site mutants appropriately displayed low Po at 150 nM cytosolic Ca^2+^ (Fig. 5); however, at 10 μM [Ca^2+^]_*cyt*_, WT RyR1 channels were activated (Fig. 5A), whereas E3893A, E3893D, E3967A, and E3967D mutant channels were not activated (Fig. 5B–F). Furthermore, 10 mM cytosolic Ca^2+^ inhibited RyR1 WT channels, but the Ca^2+^-binding site mutants were not inhibited (Fig. 5B–F). Thus, the Ca^2+^-binding site mutants were insensitive to both Ca^2+^-dependent activation at [Ca^2+^]_*cyt*_ below 100 μM, and Ca^2+^-dependent inhibition at [Ca^2+^]_*cyt*_ above 1 mM (Fig. 5B–F). Furthermore, these data suggest that a single Ca^2+^-binding site confers both the high and low affinity [Ca^2+^]_*cyt*_ dependence of RyR1 channel activity.

### ***RYR1***-RD-associated mutations near ATP and Ca^2+^ binding sites

**Fig. 6:**
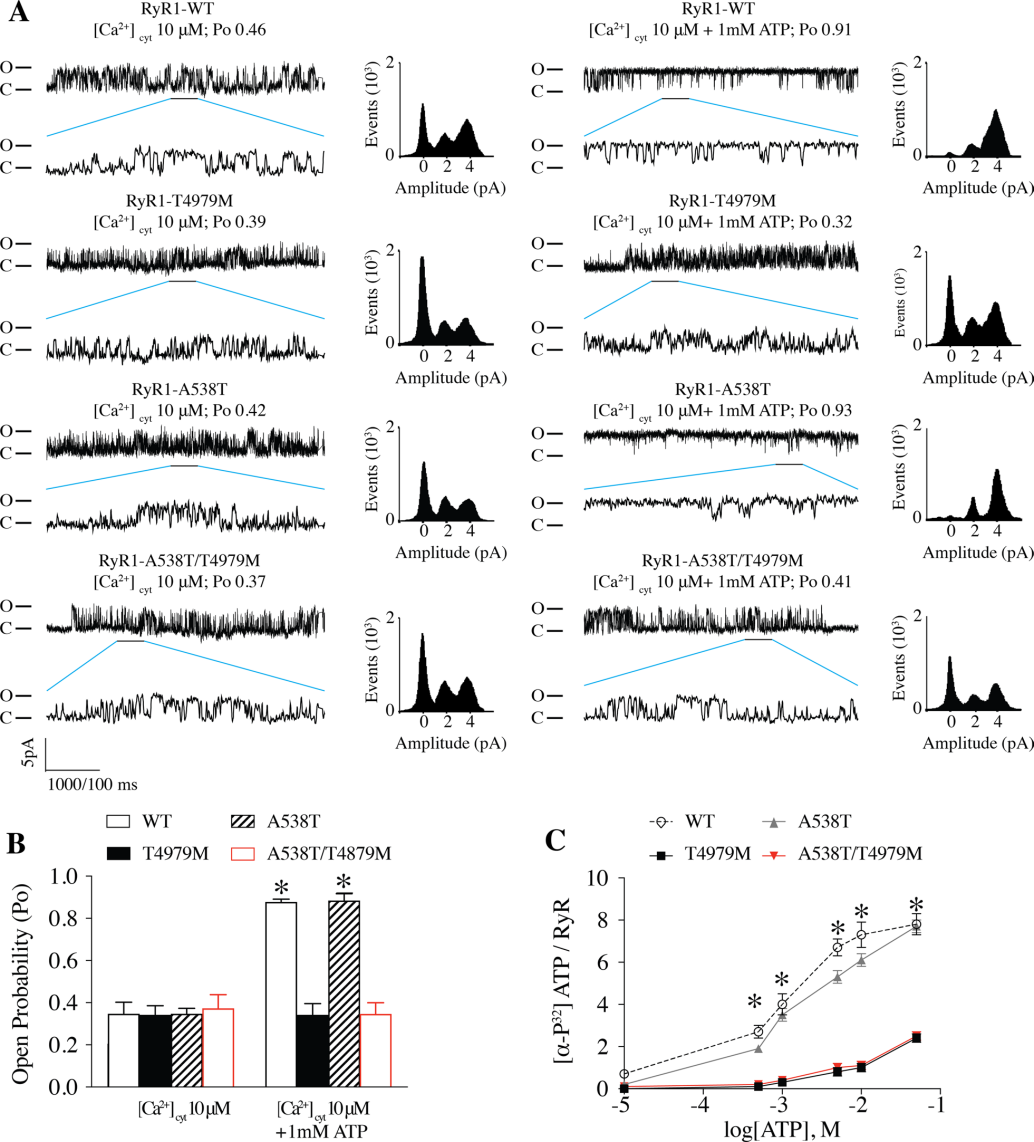
ATP binding and single channel analyses of the RyR1-RM patient-related ATP binding site mutants. **A** [α-32^P^]-ATP/ [^3^H]-ryanodine binding to ER microsomes of HEK293 cells expressing WT RyR1, T4979M, A538T and A538T/T4979M. **B** Single channel analysis of WT RyR1, T4979M, A538T and A538T/T4979M (bottom) under 10 µM Ca^2+^ only (left) or 10 µM Ca^2+^ and 1 mM ATP (right). **C** [^3^H]-ryanodine binding to ER microsomes of HEK293 cells expressing WT RyR1, T4979M, A538T and A538T/T4979M. Data are presented as mean ± S.E.M from 6 for each group

*RYR1* mutations can cause skeletal muscle dysfunction in children and adults, resulting in a wide range of disabilities, and are the most common cause of congenital myopathy [27]. We have established an *RYR1*-RD database by assembling genetic, structural, biophysical, and clinical information from more than 2200 *RYR*1-RM affected patients [27]. This database contains an RyR1 mutation at T4980M (rabbit RyR1 T4979M) associated with congenital myopathy [23, 24, 37]. RyR1-T4980 is located in the ATP binding site where it may interact with the adenine ring of ATP (Fig. 2A). The patient in the database with this mutation also had a second mutation, A538T; however, this mutation is located in the NTD of RyR1, which is far from ligand binding sites and myopathy hotspots. To study the effects of these RyR1 myopathic mutations, T4979M, A538T and A538T/T4979M mutant channels were expressed in HEK293 cells. 1 mM ATP did not increase the Po of RyR1 T4979M and A538T/T4979M mutant channels, whereas the mutant RyR1 A538T channel responded normally to ATP (Fig. 6A). A538T channels exhibited similar ATP-dependent activation as WT RyR1 channels (Fig. 6B). RyR1 T4979M and A538T/T4979M mutant channels also displayed significantly decreased [α-^32^P]-ATP binding compared to WT RyR1 channels, while RyR1 A538T exhibited normal ATP binding (Fig. 6C), Taken together, these results show that mutation of threonine to methionine at 4980 of human RyR1 significantly attenuates ATP binding and activation of the RyR1 channel. Impaired ATP-dependent activation of RyR1 may contribute to muscle weakness in *RYR*1-RD affected patients.

**Fig. 7:**
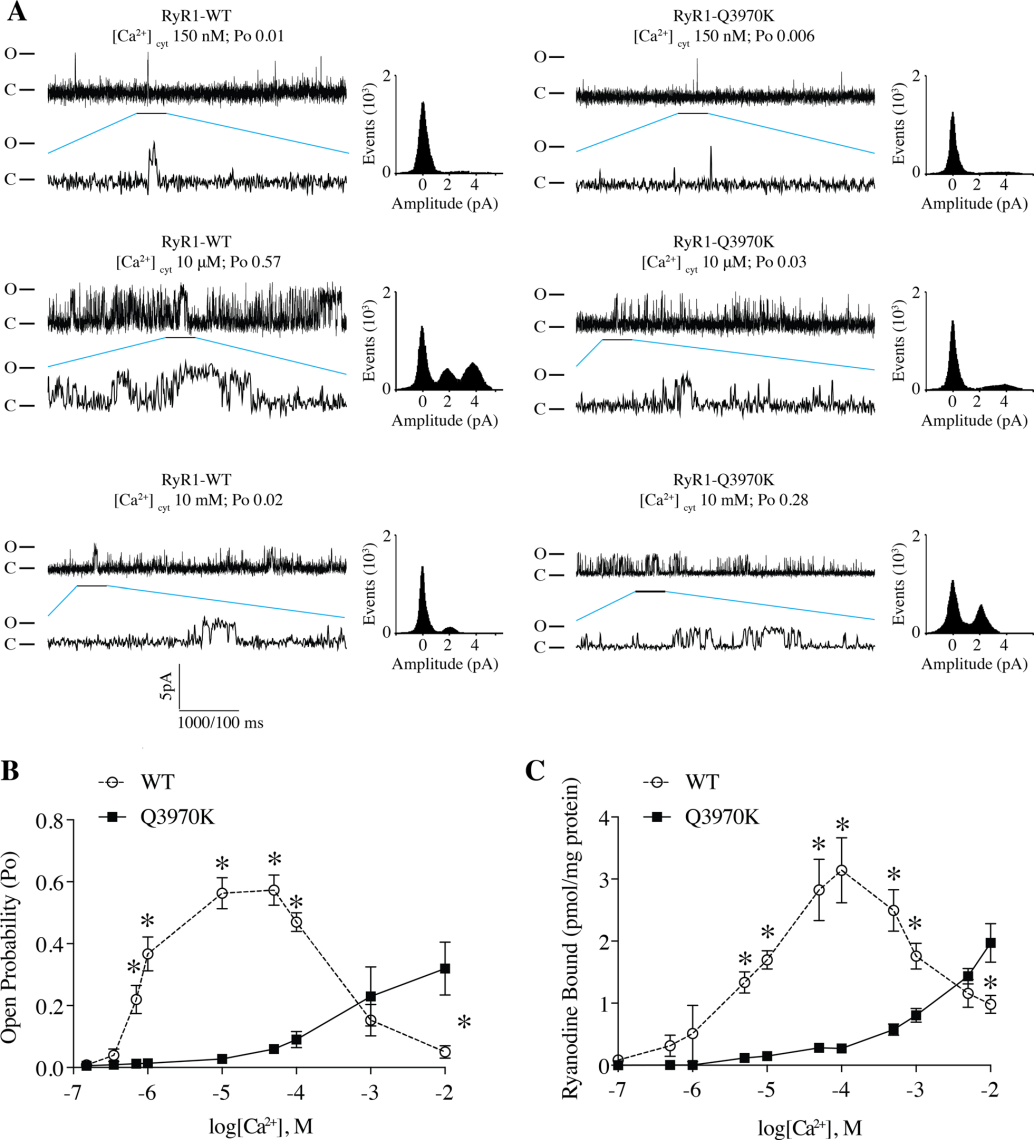
Single channel analysis and [^3^H]-binding of the RyR1-RM patient-related Ca^2+^ binding site mutants. **A** Representative single channel traces of WT RyR1 and Q3970K under 150 nM Ca2 + , 10 µM Ca^2+^, or 10 mM Ca^2+^. **B** Po versus Ca^2+^ concentration curve. **C** [^3^H]-ryanodine binding to ER microsomes of HEK293 cells expressing WT RyR1 and Q3970K mutant. Data are presented as mean ± S.E.M from 6 single channels for each group

There are also *RYR1*-RD associated mutations near the Ca^2+^-binding site, including the human RyR1 mutation Q3969K (rabbit Q3970K) and S4028L. The Q3970K mutation is linked to a form of *RYR1*-RD formerly referred to as multi-minicore disease [62]. Q3970K mutant channels displayed a right shift in Ca^2+^ dependent activation measured using single channel determinations and [^3^H]-ryanodine binding (Fig. 7A–C). Our previous work regarding the S4028L mutation showed that the RyR1 channel from this patient’s muscle biopsy exhibited elevated Po at low, non-activating [Ca^2+^]_*cyt*_, consistent with a leaky RyR1 channel that likely plays a role in the patient’s muscle weakness [27]. To assess the role of posttranslational modifications in determining the leaky behavior of disease-associated mutant RyR1 channels, RyR1-S4028L patient muscle lysates were treated with protein phosphatase 1 (PP1) and the reducing agent dithiothreitol (DTT), to reverse PKA phosphorylation and oxidation of the channel. Following this treatment, the mutant RyR1-RD linked RyR1-S4028L channels still exhibited increased sensitivity to Ca^2+^-dependent activation and showed increased activity at very low non-activating [Ca^2+^] ~ 150 nM, which is consistent with channel leak, albeit to a lesser extent than the phosphorylated and oxidized mutant channels (Additional file 1: Figure 1). These data indicate that the RyR1-RD linked mutation alone increases the sensitivity of the channel to Ca^2+^-dependent activation rendering the channel leaky and that stress-induced posttranslational modifications further exacerbate the channel dysfunction and resultant leak.

## Discussion

We previously solved the structure of RyR1 to near-atomic resolution using cryo-EM and identified ATP and Ca^2+^ binding sites [14, 76]. In the present study, we have characterized the function of the ATP and Ca^2+^ binding sites of RyR1 using mutagenesis and measurements of channel activity. Importantly, the *RYR1*-RD linked mutation T4980M, which is located in the ATP binding site, impairs ATP binding and ATP-dependent activation of the RyR1 channel. The *RyR1*-RD linked mutation Q3969K, which is located in the Ca^2+^ binding site, abolishes Ca^2+^-dependent activation. These findings suggest that interference with Ca^2+^- and ATP-dependent regulation of RyR1 may contribute to the pathophysiology of *RyR1*-RD including muscle weakness.

In skeletal muscle, the cytosolic ATP concentration is about 5 mM [25], but under physiological conditions, ATP regulation of RyR1 is influenced by Mg^2+^, a potent inhibitor of the RyR [13], and most cellular ATP is present as MgATP. Other nucleotides, such as CTP, GTP, ITP, and UTP, have no effect on RyR activity [46], which is consistent with our finding that the adenine base binding site is required for ATP binding to the RyR. We showed that T4979 is required for proper ATP-dependent regulation of RyR1 as RyR1-T4979F shows no binding with [α-^32^P] ATP. It is possible that the introduction of phenylalanine with a bulky hydrophobic group in the binding site of the adenine of ATP (T4979F) prevents the entry of ATP into that site, thus explaining why ATP binding to the T4979F mutant RyR1 is reduced. Other adenine nucleotides such as ADP, AMP, or adenosine can also increase the Po of RyR1, but with reduced efficacy [10, 30] suggesting that the phosphate groups of ATP are required for robust activation of RyR1. Previous work has reported that the triphosphate groups are the most important element for inducing a long open state in RyR2 [35], in the present study we have extended these studies by presenting data identifiying the specific amino acid residues in RyR1 that are responsible for binding to the triphosphate tail of ATP in it’s binding site. Indeed, in the present study, we demonstrate the structural basis for the critical role of the triphosphate tail of ATP in the activation of the RyR as the K4211S/K4214S/R4215S mutant RyR1, which replaces the positively charged lysine and arginine with a neutral serine, disrupts ATP-dependent activation of RyR1. The reduction in positively charged residues is presumed to decrease binding to the triphosphate tail of ATP in a way that mimics the reduced interaction of ADP and AMP with RyR1, both of which are much weaker activators of the channel [10, 30]. Since ATP is always present at mM levels in muscle it is reasonable to hypothesize that its binding to RyR1 is required for robust activation of the channel by Ca^2+^. The mutant channels which cannot bind ATP are likely less active and may contribute to impaired muscle contractility and weakness in RYR1-RD patients.

Global cytosolic [Ca^2+^]_cyt_ in resting cells is approximately 100–150 nM and rises to at least 1 µM following Ca^2+^ release through RyR1 during EC coupling. Mutations of RyR1 residues E3893 and E3967 to either A or D significantly reduced the high affinity Ca^2+^-dependent-activation of RyR1 compared to the WT channel (Fig. 4), which is consistent with previous work showing that mutation of those 2 Glutamic acid residues to Glutamine (Q) or Valine (V) interfered with Ca^2+^ regulation of the channel [72]. However, unexpectedly, these same mutations also prevented the low affinity Ca^2+^-dependent deactivation of RyR1. This finding suggests that the Ca^2+^-dependent activation and inhibition involves a single Ca^2+^ binding site. One possible mechanism to explain this phenomenon is that at low Ca^2+^ concentration (from nM to µM), this Ca^2+^ binding site was occupied by one Ca^2+^, which forms a brige between the CTD and CSol to stabilize the open state of the RyR1 channel, whereas at high Ca^2+^ concentration (mM), CTD and CSol each bind to one Ca^2+^, which disrupts the CTD-CSol interface to make the channel close. A patient with a Q3970K mutation at this site exhibited the same impaired Ca^2+^-dependent activation and deactivation as the E3893A/D and E3967A/D mutant channels, which is consistent with a previous study showing that RyR1-Q3970K displayed low Ca^2+^ dependent channel activity [11]. It is likely that the additional positive charge from the lysine substitution in the Q3970K mutant channel reduces Ca^2+^-binding at this site. Similarly, the replacement of glutamic acid at 3967 or 3893 with the neutral alanine may reduce the affinity of the Ca^2+^ and thus impair both activation and deactivation, indicating the negative charges of glutamic acid are critical for the Ca^2+^ binding of RyR1. Replacing the glutamic acid at 3967 with an aspartic acid preserves the negative charge; however, since the side chain of glutamate is larger than that of aspartic acid, the interaction with Ca^2+^ may be weakened.

A previous study suggested that E4032 is part of the Ca^2+^ binding site of RyR1, as mutation of E4032 reduced Ca^2+^ activation in both RyR1 and RyR2 [20, 34]. However, our cryo-EM RyR1 structure indicates that E4032 is not close enough to the Ca^2+^ binding site to form a direct interaction with the bound Ca^2+^. Nevertheless, it may stabilize the CTD-CSol interface via hydrogen bonding to the amide nitrogens at the end of one of the CTD helices [14]. Interestingly, a mutant channel RyR1-S4028L which has been linked to *RYR1*-RM [27], exhibited increased Ca^2+^-dependent activation of RyR1 at low non-activating [Ca^2+^]_*cyt*_, which is consistent with channel leak [27], suggesting that the polar side chain of serine is necessary to stabilize the CTD-CSol interface. Moreover, blocking or reversing post-translational modifications of RyR1 (PKA phosphorylation and oxidation) revealed that the channel mutation alone is sufficient to cause Ca^2+^ leak and that the posttranslational modications are additive in terms of leak.

The present study identifies functional ATP and Ca^2+^-dependent regulatory sites in RyR1. Moreover, these are also the sites of *RyR1*-RM disease causing mutations, indicating that defective regulation of RyR1 by Ca^2+^ and ATP may be a component of the pathophysiology of this form of myopathy.

## Materials and methods

### Ryanodine receptor mutagenesis and expression

The recombinant RyR1 constructs T4979F, K4211S/K4214S/R4215S, E3893A, E3893D, E3967A, E3967D, T4979M, and Q3970K were generated by introducing the respective mutations into fragments of rabbit RyR1 using QuikChange II XL Site-Directed Mutagenesis Kit (Agilent). Each fragment was subsequently subcloned into a full length RyR1 construct in pcDNA3.1 vector and confirmed by sequencing. The primers used to introduce specific mutants (codons in parentheses, mutated nucleotides in bold) are as follows: 5′-cacggcttcgagacccac(**ttc**)ctagaggagcacaatctg for T4979F, 5′-gtgggagatgccccaggtc(a**gc**)gagtcc(a**gc**)(**a**gc)cagttcatcttc for K4211S-K4214S-R4215S, 5'-gcagctgctctgt(g**c**g)gggcacaacaacg for E3893A, 5′-ctgcagctgctctgt(ga**c**)gggcacaac for E3893D, 5′-caacagcctcacc(g**c**g)tacatccagggcc for E3967A, 5′-acagcctcacc(ga**c**)tacatccagggcc for E3967D, 5′-cacggcttcgagacccac(a**t**g)ctagaggag for T4979M, and 5′-ctcaccgagtacatc(**a**ag)ggcccctgcac for Q3970K. For all mutants, the second primer was the complementary reverse to the forward primer. HEK293 cells grown in DMEM supplemented with 10% (vol/vol) FBS (Invitrogen), penicillin (100 U/mL), streptomycin (100 µg/mL), and L-glutamine (2 mmol/L) were transfected with WT or mutant RyR1 cDNA using Lipofectamine 2000 (ThermoFisher Scientific). Cells were collected 48 h after transfection.

### ER vesicles preparation

ER vesicles from HEK293 cells expressing WT or mutant RyR1 were prepared by homogenizing cell pellets on ice using a Teflon-glass homogenizer with two volumes of solution containing 20 mmol/L (mM) Tris-maleate (pH 7.4), 1 mM EDTA, 1 mM DL-Dithiothreitol (DTT) and protease inhibitors (Roche). Homogenate was then centrifuged at 4,000 xg for 15 min at 4 °C and the resulting supernatant was centrifuged at 40,000 xg for 30 min at 4 °C. The final pellet, containing the ER fractions, was resuspended and aliquoted in 250 mM sucrose, 10 mM MOPS (pH 7.4), 1 mM EDTA, 1 mM DTT and protease inhibitors. Samples were frozen in liquid nitrogen and stored at −80 °C.

### SR microsome preparation

Skeletal muscle SR microsomes were prepared as previsouly described [27]. Briefly, muscle samples were homogenized on ice using a Teflon-glass homogenizer with 2 volumes of: 20 mmol/L (mM) Tris-maleate (pH 7.4), 1 mM EDTA, 1 mM DL-dithiothreitol (DTT) and protease inhibitors (Roche). The resulting homogenate was then centrifuged at 4,000 g for 15 min at 4 °C and the supernatant was centrifuged at 50,000 g for 45 min at 4 °C. Pellets were resuspended in lysis buffer containing 300 mM sucrose.

### [^3^H] Ryanodine and [α-^32^P]-ATP binding

Skeletal muscle SR microsomes or ER vesicles from HEK293 cells expressing WT or mutant RyR1 were incubated in media containing 5 nM [^3^H]-ryanodine or 5 nM [α-^32^P]-ATP, 1 M NaCl, 20 mM HEPES, and 0.5 mM EGTA at 37 °C for 2 h. The concentration of free Ca^2+^ was calculated with WinMaxC (version 2.50; www.stanford.edu/~cpatton/maxc.html). For ATP activation, 1 mM ATP and 1 mM free Ca^2+^ were added during incubation. The binding mix was then filtered through Whatman GF/B filters presoaked with 1% polyethyleneimine. The filters were washed three times with 5 mL of ice-cold washing buffer containing 0.2 M NaCl and 5 mM HEPES (pH 7.5) to remove unbound [^3^H]-ryanodine, and the amount of remaining [^3^H]-ryanodine was determined by liquid scintillation counting. Nonspecific binding was determined by measuring [^3^H]-ryanodine binding in the presence of 10 μM unlabeled ryanodine. All binding assays were done in duplicate.

### Single-channel recordings

ER vesicles were fused to planar lipid bilayers formed by painting a lipid mixture of phosphatidylethanolamine and phosphatidylcholine (Avanti Polar Lipids) in a 5:3 ratio in decane across a 200 µm hole in polysulfonate cups (Warner Instruments) separating two chambers. The *trans* chamber (1.0 mL), representing the intra-SR (luminal) compartment, was connected to the head stage input of a bilayer voltage clamp amplifier. The *cis* chamber (1.0 mL), representing the cytoplasmic compartment, was held at virtual ground. Asymmetrical solutions used were as follows for the *cis* solution: 1 mM EGTA, 250/125 mM Hepes/Tris, 50 mM KCl, 0.64 mM CaCl_2_, pH 7.35; and for the trans solution: 53 mM Ca(OH)_2_, 50 mM KCl, 250 mM Hepes, pH 7.35. The concentration of free Ca^2+^ in the *cis* chamber was calculated as previously described [14]. ER vesicles were added to the *cis* side and fusion with the lipid bilayer was induced by making the *cis* side hyperosmotic by the addition of 400–500 mM KCl. After the appearance of potassium and chloride channels, the *ci*s side was perfused with the *c*is solution. At the end of each experiment, 10 µM ryanodine was added to block the RyR channel. Single-channel currents were recorded at 0 mV using a Bilayer Clamp BC-525D (Warner Instruments), filtered at 1 kHz using a Low-Pass Bessel Filter 8 Pole (Warner Instruments), and digitized at 4 kHz. All experiments were performed at room temperature (23 °C). Data acquisition was performed by using Digidata 1322A and Axoscope 10.1 software (Axon Instruments). The recordings were analyzed using Clampfit 10.1 (Molecular Devices) and Graphpad Prism software.

### Immunoprecipitation and immunoblotting

RyR1 were immunoprecipitated from extracts of human patient muscle biopsy using anti- anti-RyR1-specific antibodies (2 μg) in 0.5 ml of a modified radioimmune precipitation assay buffer (50 mm Tris–HCl, pH 7.2, 0.9% NaCl, 5.0 mm NaF, 1.0 mm Na_3_VO_4_, 1% Triton X-100 and protease inhibitors) overnight at 4 °C as previously described [2]. The immune complexes were incubated with protein A-Sepharose beads (Sigma-Aldrich) at 4 °C for 1 h and the beads were washed three times with the modified radioimmune precipitation assay buffer. The immunoprecipitated proteins were size-fractionated on SDS–polyacrylamide gels (4–20% for RyR1) and transferred to nitrocellulose membranes for 2 h at a current of 200 mA. Immunoblots were probed with the following primary antibodies: anti-RyR1 (Affinity Bioreagents, 1:2,000 dilution), anti-Cys-NO (Sigma-Aldrich, 1:1,000 dilution), or anti-phospho-RyR-Ser(P)-2844 (Affinity Bioreagents, 1:5,000 dilution). To determine channel oxidation, the carbonyl groups in the protein side chains were derivatized to 2,4-dinitrophenol (DNP) by reaction with 2,4-dinitrophenylhydrazine. The DNP signal associated with total oxidized protein or with RyR was determined using a specific anti-DNP antibody according to the manufacturer's instructions (Millipore). All immunoblots were developed using an Odyssey system (LI-COR Biosciences), with infrared-labeled anti-mouse or anti-rabbit IgG (Abcam, 1:10,000 dilution) secondary antibodies.

### Statistics

All results are presented as the mean ± SEM. Statistical analyses were performed using the unpaired Student’s t test, 2-tailed (for 2 groups), or the 1-way ANOVA with Tukey–Kramer post hoc correction (for groups of 3 or more) unless otherwise indicated. P < 0.05 was considered to be statistically significant.

## Supplementary Information

**Additional file 1: Figure 1.** RyR1-S4028L patient mutation causes RyR1 channel leak. (**A**) The mutant RyR1-S4028L channel was PKA phosphorylated at Ser2844 and oxidized (DNP) compared to control. PP1 and DTT were used to reverse the oxidation and phosphorylation. (**B**) The mutant RyR1-S4028L channels exhibited increased sensitivity to Ca^2+^-dependent activation consistent with channel leak as determined by ^3^[H]-ryanodine binding at the indicated [Ca^2+^]_*cyt*_. Data are presented as mean ± S.E.M from 4 for each group *P < 0.05 vs. WT; ^#^*P* < 0.05 vs. RyR1-S4028L, ANOVA, Tukey-Kramer with post hoc correction.

## Data Availability

The data supporting the findings of this are documented within the paper and are available from the corresponding author upon request.
