# Supplementary material for: RyR1-related myopathy mutations in ATP and calcium binding sites impair channel regulation
Source: Acta Neuropathol Commun. 2021 Nov 22;9:186. doi: 10.1186/s40478-021-01287-3 (PMC8609856; doi:10.1186/s40478-021-01287-3)
Supplement: Supplementary file 1 — Additional file 1: Figure 1. RyR1-S4028L patient mutation causes RyR1 channel leak. (A) The mutant RyR1-S4028L channel was PKA phosphorylated at Ser2844 and oxidized (DNP) compared to control. PP1 and DTT were used to reverse the oxidation and phosphorylation. (B) The mutant RyR1-S4028L channels exhibited increased sensitivity to Ca2+-dependent activation consistent with channel leak as determined by 3[H]-ryanodine binding at the indicated [Ca2+]cyt. Data are presented as mean ± S.E.M from 4 for each group *P < 0.05 vs. WT; #P < 0.05 vs. RyR1-S4028L, ANOVA, Tukey-Kramer with post hoc correction. [file 40478_2021_1287_MOESM1_ESM.docx]

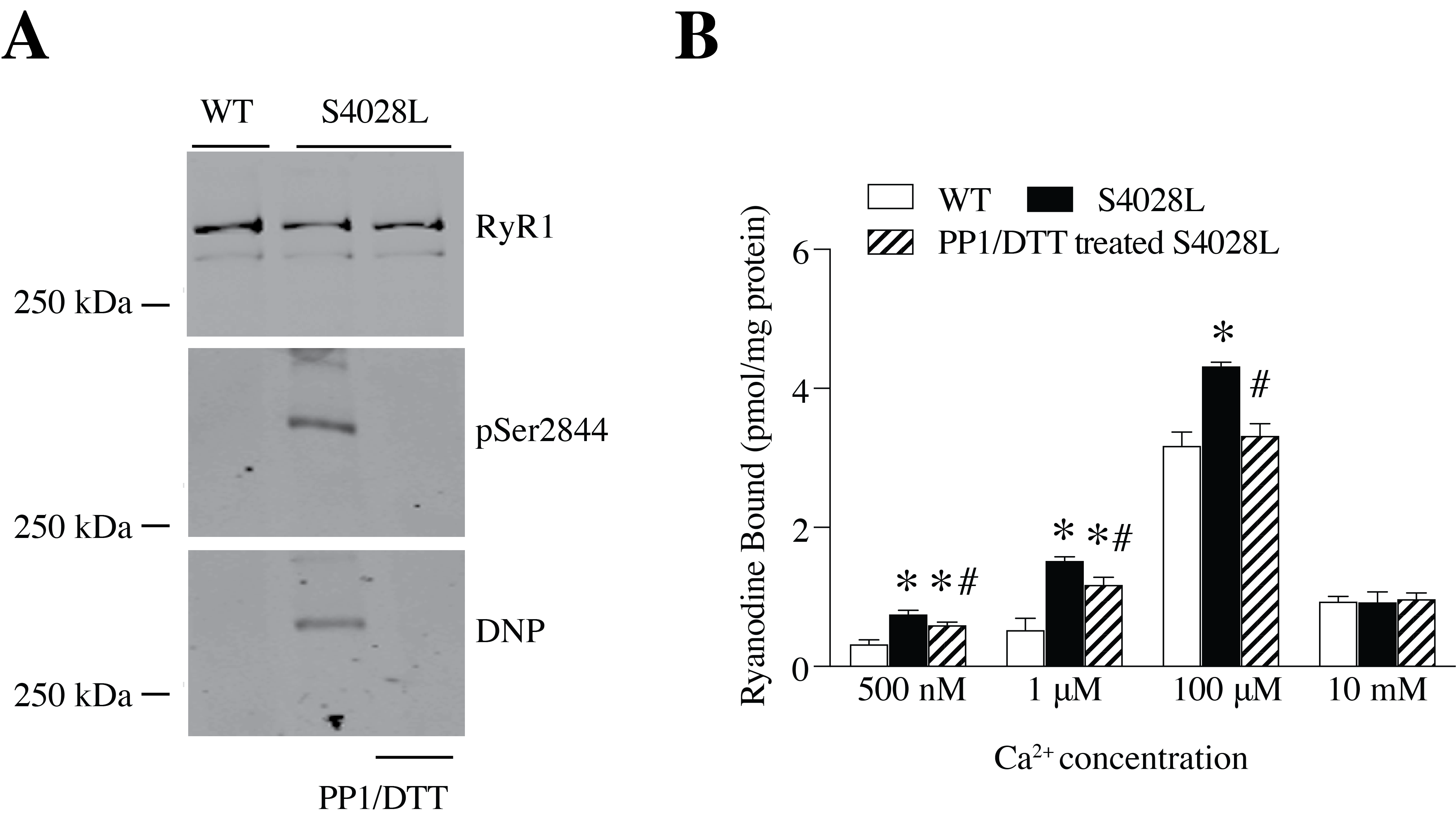


**Supplemental Figure 1. RyR1-S4028L patient mutation causes RyR1 channel leak.** (**A**) The mutant RyR1-S4028L channel was PKA phosphorylated at Ser2844 and oxidized (DNP) compared to control. PP1 and DTT were used to reverse the oxidation and phosphorylation. (**B**) The mutant RyR1-S4028L channels exhibited increased sensitivity to Ca^2+^-dependent activation consistent with channel leak as determined by ^3^[H]-ryanodine binding at the indicated [Ca^2+^]*_cyt_*. Data are presented as mean ± S.E.M from 4 for each group **P* < 0.05 vs. WT; #*P* < 0.05 vs. RyR1-S4028L, ANOVA, Tukey-Kramer with post hoc correction.
